# Functional Analyses of a Small Secreted Cysteine-Rich Protein ThSCSP_14 in *Tilletia horrida*

**DOI:** 10.3390/ijms232315042

**Published:** 2022-11-30

**Authors:** Xinyue Shu, Deze Xu, Yuqi Jiang, Juan Liang, Ting Xiang, Yuxuan Wang, Weike Zhang, Xue Han, Chunhai Jiao, Aiping Zheng, Ping Li, Desuo Yin, Aijun Wang

**Affiliations:** 1 College of Agronomy, Sichuan Agricultural University, Chengdu 611130, China; 2 Food Crop Research Institute, Hubei Academy of Agriculture Sciences, Wuhan 430064, China; 3 Rice Research Institute, Sichuan Agricultural University, Chengdu 611130, China

**Keywords:** rice, *Tilletia horrida*, effector, cell death, immune response

## Abstract

*Tilletia horrida* is a biotrophic basidiomycete fungus that causes rice kernel smut, one of the most significant diseases in hybrid rice-growing areas worldwide. Little is known about the pathogenic mechanisms and functions of effectors in *T. horrida*. Here, we performed functional studies of the effectors in *T. horrida* and found that, of six putative effectors tested, only ThSCSP_14 caused the cell death phenotype in epidermal cells of *Nicotiana benthamiana* leaves. ThSCSP_14 was upregulated early on during the infection process, and the encoded protein was secreted. The predicted signal peptide (SP) of ThSCSP_14 was required for its ability to induce the necrosis phenotype. Furthermore, the ability of ThSCSP_14 to trigger cell death in *N. benthamiana* depended on suppressing the G2 allele of Skp1 (SGT1), required for Mla12 resistance (RAR1), heat-shock protein 90 (HSP90), and somatic embryogenesis receptor-like kinase (SERK3). It is important to note that ThSCSP_14 induced a plant defense response in *N. benthamiana* leaves. Hence, these results demonstrate that ThSCSP_14 is a possible effector that plays an essential role in *T. horrida*–host interactions.

## 1. Introduction

As a biotrophic plant fungal pathogen, *Tilletia horrida* causes kernel smut disease in rice crops [1]. Infecting rice floral organs via the stigma at the flowering stage, *T. horrida*-develops powdery dark teliospore balls in the kernels when nearing the yellow ripeness stage. These teliospores are the main sources of *T. horrida*’s host-infection and spread; they possess strong durability, and their survival time exceeds 1 year in soil, with survival of more than 3 years on infected host seed [2]. The rice yield loss due to *T. horrida* can reach up to 5–20% under optimum environmental conditions [3]. Despite this crop damage, there is surprisingly little study of *T. horrida*’s pathogenic mechanism and virulence-related genes, making this important disease of rice difficult to monitor and control.

Different phytopathogens can differ in their life history in terms of how they obtain nutrients [4]. Still, whether biotrophs, hemibiotrophs, or necrotrophs, plant pathogenic fungi typically secrete large amounts of effector proteins to facilitate their infection and suppression of plant defenses during colonization process of hosts [5,6,7]. Although such effectors are crucial, only a few proteins secreted by pathogens have been confirmed to function as effectors. For example, in *Ustilaginoidea virens*, 13 secreted effectors are able to cause cell-death phenotypes in *Nicotiana benthamiana* [8]. The effector Pst_13661 suppresses Bax-induced cell death in plants and plays a crucial role in *Puccinia striiformis*–wheat interactions [9]. Several effectors have been studied in smut fungi, such as Pit2, See1, Pep1, Cmu1, and Tin2 in *Ustilago maydis* [10,11,12,13,14]. Of those, Pep1 figures prominently in the process by which *U. maydis* and the covered smut of barley fungus *U. hordei* each penetrate the host, and have a conserved function in establishing host smut pathogen interaction [12]. For *T. horrida*, only two effectors—smut_5844 and smut_2965—have been identified as capable of triggering non-host cell death and an immune response [15].

The recognition between receptor proteins and effectors could suppress plant defense responses, thereby facilitating pathogen infection. For example, the effector SsSSVP1 in *Sclerotinia sclerotiorum* interacts with QCR8, a subunit of the cytochrome b-c1 complex of the mitochondrial respiratory chain, to markedly promote that pathogen’s infection of hosts [16]. In *P. striiformis*, the effector Pst_12806 can interfere with the photosynthesis of wheat by interacting with the photosynthesis-related protein TaISP, further reducing the ROS (reaction oxygen species) burst and plant-defense-related gene expression mediated by chloroplast, thereby augmenting that pathogen’s infection [17]. Conversely, the activation of plant immune response is related to interactions between receptor proteins and certain effectors, such as the receptor-like proteins Cf-4, Cf-2, Cf-9, and Cf-4E in tomato that, respectively, interact with the effectors Avr4, Avr2, Avr9, and Avr4E in *Cladosporium fulvum* [18,19].

Analysis of *T. horrida*’s genome sequence data revealed that the JY-521 genome encodes 597 secreted proteins, some of which could be effectors [20]. However, only two secreted proteins, namely smut_2965 and smut_5844, which trigger necrosis and defense response in non-host plants, are known to function as effectors [15]. In addition, a signal peptide (SP) sequence was needed for smut_5844 to induce non-host cell death, whereas ribonuclease catalytic active sites were crucial for smut_2965. In the present study, six candidate effectors in the genome of *T. horrida* JY-521 [20] were assessed for their activity in triggering cell death using a transient expression system in leaves of *N*. *benthamiana*. The candidate effector protein smut_3787 (ThSCSP_14) induced cell death in epidermis cells of tobacco leaves. The predicted SP sequence of ThSCSP_14 is shown to be necessary for triggering the necrosis phenotype. We further found that suppression of the G2 allele of Skp1 (SGT1), the latter required for Mla12 resistance (RAR1), heat-shock protein 90 (HSP90), and somatic embryogenesis receptor-like kinase (SERK3) is a prerequisite for ThSCSP_14 to trigger cell death in *N. benthamiana*. In addition, ThSCSP_14 activated plant defense responses in *N. benthamiana*. Altogether, these findings advance our understanding of molecular mechanisms underpinning rice–*T. horrida* interactions.

## 2. Results

### 2.1. ThSCSP_14 in T. horrida Induces Cell Death in N. benthamiana

**Figure 1:**
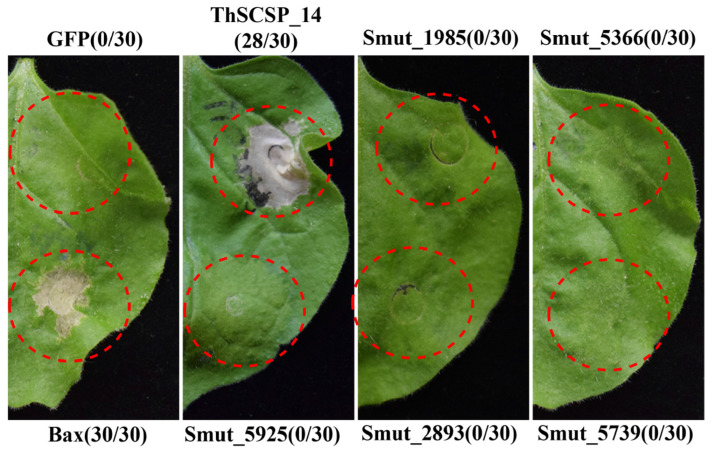
Putative effectors in *Tilletia horrida* trigger necrosis phenotype in epidermis cell of tobacco leaves. ThSCSP_14 and positive control BAX protein triggered cell death in epidermis cell of tobacco leaves; however, other five selected proteins and negative control green fluorescent protein (GFP) did not. The 30/30 denotes 30 of 30 infiltrated tobacco leaves exhibiting the necrosis phenotype. Representative photos were taken at 4 days post-infiltration (dpi).

We had previously predicted 597 potential secreted proteins from the sequenced genome of *T. horrida* [20]. To identify effector proteins of this pathogen, we focused on six genes that encode small cysteine-rich secreted proteins (SCSPs) which had a N-terminal SP but lacking a transmembrane domain, which were predicted to be effectors on http://effectorp.csiro.au/ (accessed on 15 May 2022) [21]. We investigated the ability of these six SCSPs to induce cell death by transient expression in *N. benthamiana* leaves using *Agrobacterium*-mediated methods. These results showed that only positive control Bax and ThSCSP_14 triggered cell death in *N. benthamiana* leaves at 4 days post-inoculation. However, a negative control of the green fluorescent protein (GFP) and other five SCSPs constructs did not trigger cell death (Figure 1).

### 2.2. ThSCSP_14 Is Unique to Tilletia fungi

**Figure 2:**
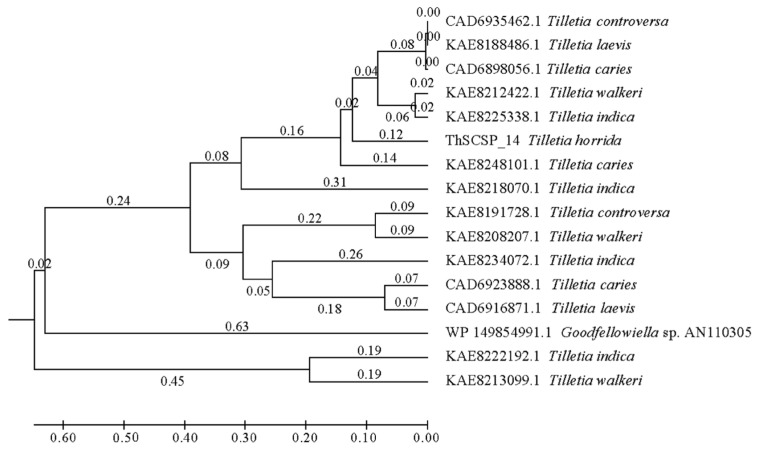
Derived phylogeny of ThSCSP_14 and its homology-secreted proteins.

ThSCSP_14 encoded a 429 aa protein that contained a predicted N-terminal SP (22 aa), but no conserved domain could be found. According to previous research, the effector proteins of plant pathogenic fungi are supposed to be highly conserved [22]. Interestingly, we only found homolog genes of ThSCSP_14 in several Tilletia fungi, namely *T. indica*, *T. walkeri*, *T. controversa*, and *T. laevis* (Figure 2). This result revealed that the potential SCSP ThSCSP_14 is unique to Tilletia fungi, and these smut fungi are important pathogens of rice and wheat. Hence, we speculated that ThSCSP_14 play a specific role in rice–*T. horrida* interactions.

### 2.3. Functional Validation of the Predicted SP of ThSCSP_14

**Figure 3:**
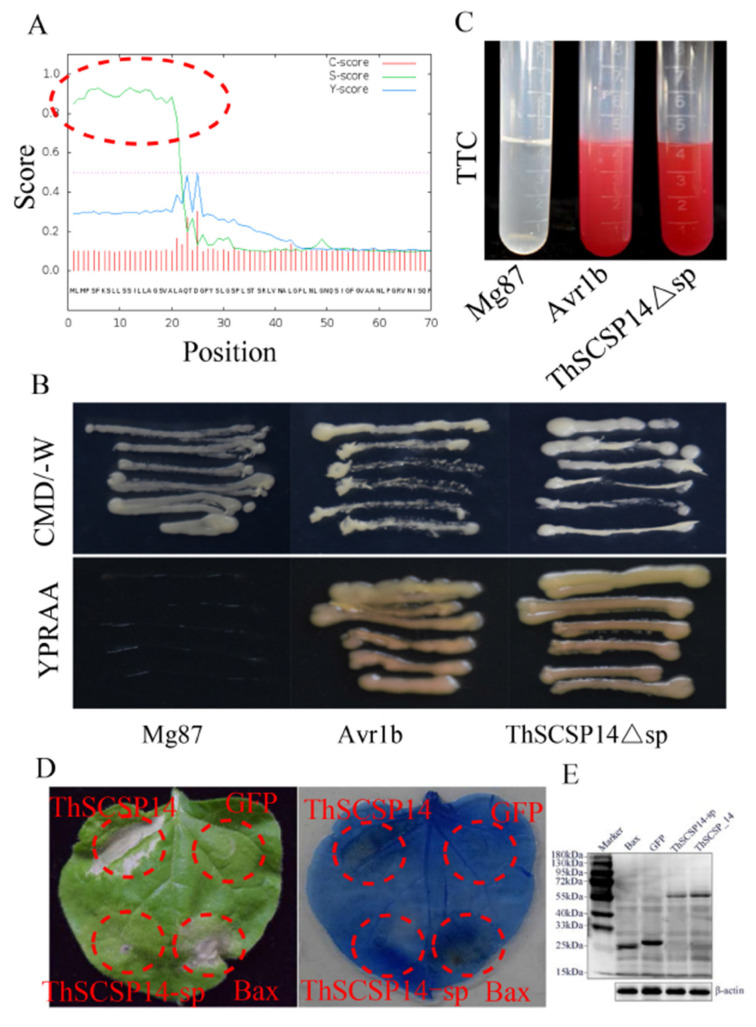
Functional analysis of ThSCSP_14′s signal peptide (SP) sequence (ThSCSP_14Δsp). (**A**) The SP sequence was predicted in ThSCSP_14. (**B**) The yeast invertase secretion assay was used to identify the secretory function of ThSCSP_14Δsp. To cultivate the transformed YTK12 yeast strains, the CMD-W and YPRAA media with raffinose as the sole carbon source were each used. The positive control is SP sequence of *Phytophthora sojae* Avr1b; the and negative control is the SP sequence of *Magnaporthe oryzae* Mg87. (**C**) Enzymatic activity of invertase was identified by performing the 2, 3, 5-triphenyltetrazolium chloride experiment. The positive control is the SP sequence of *P. sojae* Avr1b; the negative control is the SP sequence of *M. oryzae* Mg87. (**D**) ThSCSP_14Δsp is required to induce cell death. (**E**) The protein’s expression in *Nicotiana benthamiana* observed by Western blot experiments. ThSCSP_14-sp denoted the sequence of ThSCSP_14 without SP.

The N-terminal 1–22 aa of ThSCSP_14 was predicted to be a SP (Figure 3A). We used a yeast secretion assay to verify the secretory function of the predicted SP of ThSCSP_14 (ThSCSP_14Δsp). This result showed that the fusion protein with ThSCSP_14Δsp and positive control (fusion protein with the secretion signal of *Phytophthora sojae* Avr1b) was secreted from the transformed yeast YTK12 (Figure 3B). However, as a negative control, the N-terminus of Mg87 in *M. oryzae* was not secreted from the transformed yeast YTK12 (Figure 3A) [23]. Additionally, the function of ThSCSP_14Δsp was also identified by the color reaction of 2, 3, 5-triphenyltetrazolium chloride (TTC). The secreted invertase can reduce the colorless TTC into insoluble red 1, 3, 5-triphenylformazan (TPF) (Figure 3C). These results indicated that the predicted ThSCSP_14Δsp leads to the secretion of invertase and functionally secreted proteins. Furthermore, the SP of multiple pathogenic fungi effectors is important for inducing cell death in plants [24]. To clarify the function of ThSCSP_14Δsp in induced cell death, we performed transient expression assays of ThSCSP_14 with and without the SP in *N. benthamiana* leaves. ThSCSP_14-sp (i.e., ThSCSP_14 lacking the SP) no longer induced cell death (Figure 3D). Western blot experiments showed ThSCSP_14-sp expressed in the infiltrated leaves of *N. benthamiana* (Figure 3E). These results demonstrated that SP is required for ThSCSP_14 to trigger cell death in *N. benthamiana*.

### 2.4. ThSCSP_14 Expression during T. horrida Infection

**Figure 4:**
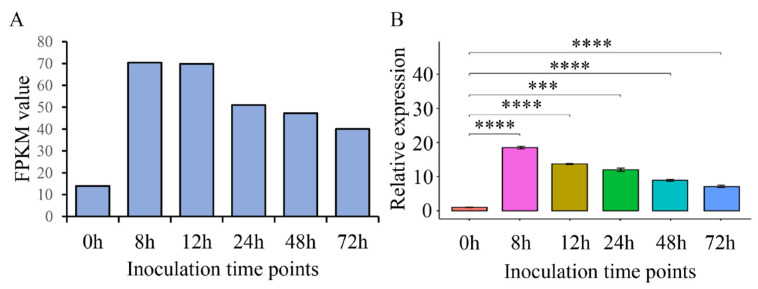
Expression of ThSCSP_14 during *T. horrida* infection of the kernel smut–susceptible rice cultivar 9311A. (**A**) Transcriptome data. (**B**) The qRT-PCR analysis of ThSCSP_14. Rice kernels inoculated against *T. horrida* were collected at 0, 8, 12, 24, 48, and 72 h post-inoculation for gene expression analyses using the quantitative real-time reverse transcription polymerase chain reaction. *UBQ* expression served as an internal reference for normalizing expression levels within the samples. Error bars are the standard deviation (SD) of four independent replicates (*** *p* < 0.001, **** *p* < 0.0001).

Plant pathogen effectors are induced and their expression upregulated in the interaction process between a pathogen and its host [25]. The expression levels of ThSCSP_14 were upregulated at different post-inoculation times (8, 12, 24, 48, 72 h) according to transcriptome data (Figure 4A) [20]. To further observe the expression pattern of ThSCSP_14 during *T. horrida* infection, we used *T. horrida* strain JY-521 to infect the rice cultivar 9311A, which is susceptible to *T. horrida*. The expression levels of ThSCSP_14 at 8, 12, 24, 48, and 72 h were detected by qRT-PCR (quantitative real-time-polymerase chain reaction). We found that ThSCSP_14 was induced and its expression upregulated after *T. horrida* inoculation (Figure 4B), a result that indicated ThSCSP_14 has critical roles in *T. horrida*–rice interactions.

### 2.5. ThSCSP_14-triggered Cell Death in N. benthamiana Depends on SGT1, RAR1, HSP90, and SERK3/Bak1

**Figure 5:**
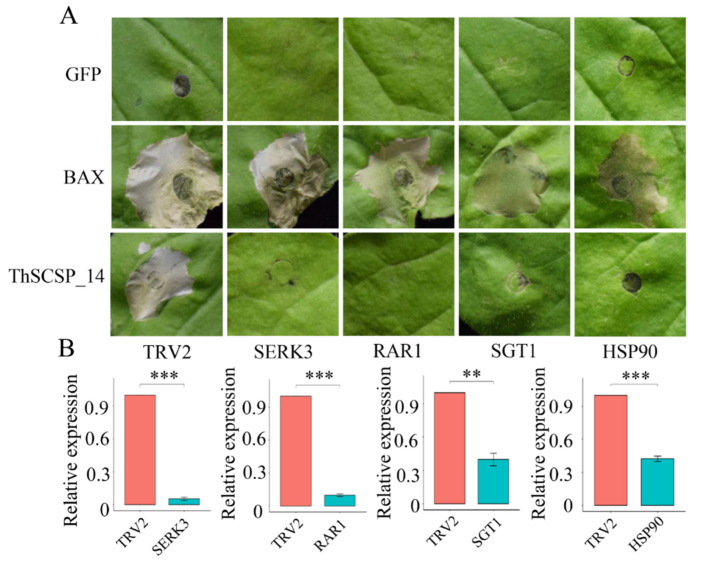
The RAR1, SGT1, HSP90, and SERK3 are necessary for the necrosis phenotype triggered by ThSCSP_14. (**A**) Representative photographs of the ThSCSP_14-triggered necrosis phenotype in silenced *Nicotiana benthamiana* leaves at 4 days post-infiltration (dpi). *Agrobacterium tumefaciens* carrying ThSCSP_14 was infiltrated into the upper leaves of silenced plants at 21 dpi using tobacco rattle virus (TRV) constructs. The control was un-silenced plants. (**B**) The transcript expression levels of RAR1, SGT1, HSP90, and SERK3 in the corresponding silenced plants analyzed by qRT-PCR. The constitutively expressed gene *NbActin* served as internal reference. Error bars are the standard deviation SD of three biological replicates (** *p* < 0.01; *** *p* < 0.001).

*SGT1*, *HSP90*, and *RAR1* are important in the host-defense response induced by R protein [26,27]. Thus, we determined the functioning of these three genes in cell death induced by ThSCSP_14 by limiting their expression in *N. benthamiana* via virus-induced gene silencing (VIGS). The results showed that ThSCSP_14 is incapable of inducing cell death in the SGT1-, HSP90-, and RAR1-silenced plants (Figure 5A); however, the necrosis phenotype was observed in these silenced plants after injecting the positive control Bax protein. These results proved that these three genes are essential for cell death mediated by ThSCSP_14. The receptor-like kinase SERK3/Bak1 is a crucial factor involved in pathogen-associated molecular pattern (PAMP) of plants [28]. To clarify whether ThSCSP_14 is in association with PAMP-triggered immunity (PTI) reaction, we also detected the cell death in SERK3/Bak1-silenced *N. benthamiana* plants after their ThSCSP_14 infiltration. These results indicated that ThSCSP_14 also failed to elicit the cell-death phenotype in SERK3/Bak1-silenced *N. benthamiana* plants (Figure 5A). We then used qRT-PCR to verify the silenced expression of SGT1, HSP90, RAR1, and SERK3/Bak1 in *N. benthamiana* plants (Figure 5B). Therefore, ThSCSP_14 is also involved in the PTI immune reaction response to SERK3/Bak1 in *N. benthamiana*.

### 2.6. Subcellular Localization of ThSCSP_14

**Figure 6:**
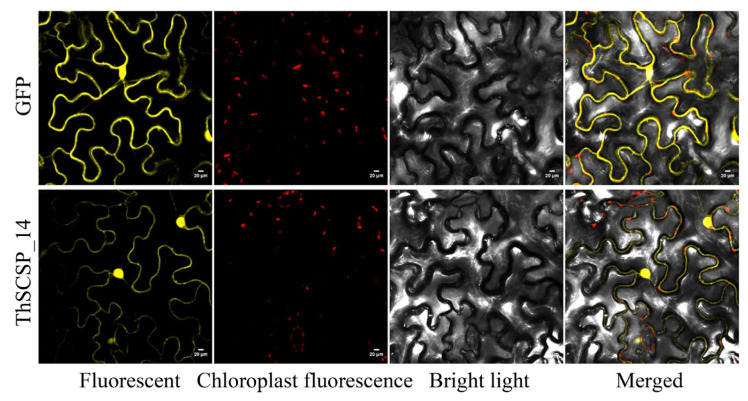
Subcellular localization of ThSCSP_14 transiently expressed in *Nicotiana benthamiana* leaves. The vector PHB carrying GFP was the control. ThSCSP_14 and GFP fluorescence signals were visualized using laser confocal fluorescence microscopy at 48 h after infiltration and were depicted in yellow. The red dots denoted the chloroplast channel. The white scale bars represented 20 μm.

Tobacco (N. *benthamiana*) can be genetically transformed with good efficiency and is amenable to transient protein expression. It is widely used in studies of proteins’ localization and interaction and in plant-based systems for protein expression and purification [29]. Thus, to detect the subcellular localization of ThSCSP_14 in the plant cell, 2, 35S: ThSCSP_14-sp-GFP was transiently expressed in tobacco leaf epidermis cells, with fluorescence observed by confocal microscopy. Transient expression of 2 × 35S: GFP was used as a marker. The ThSCSP_14-sp-GFP protein was detected in the cytoplasm and nucleus of the transiently transformed tobacco leaf epidermis cells (Figure 6).

### 2.7. ThSCSP_14 Triggers Plant Immunity Responses

**Figure 7:**
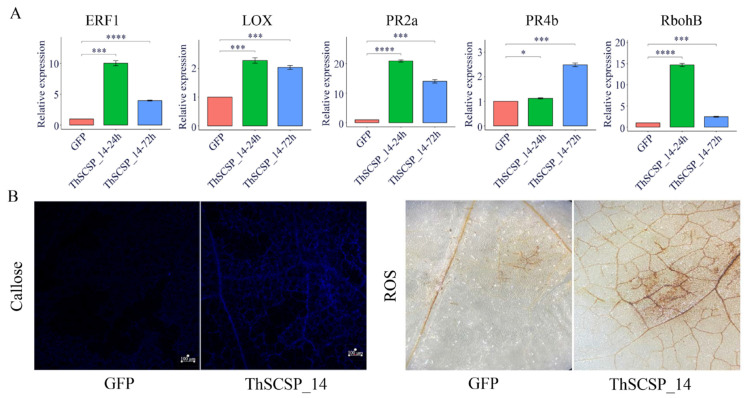
ThSCSP_14 triggers immunity responses in *Nicotiana benthamiana*. (**A**) Expression levels of genes related to plant immunity in *N. benthamiana* leaves were detected at 24 and 72 h post-inoculation. Error bars are the standard deviation (SD) of four independent replicates (* *p* < 0.05; *** *p* < 0.001; **** *p* < 0.0001). (**B**) Accumulation of reactive oxygen species (ROS) and deposition of callose in *N. benthamiana*. Bars = 100 μm. These experiments were replicated three times, using six leaves per biological replicate.

In order to determine whether ThSCSP_14 activates a plant defense response, we detected the expression of several defense-related genes in *N. benthamiana* after ThSCSP_14 was transiently expressed, using qRT-PCR. These results indicated that, in comparison with GFP alone, transcription factor ERF1 (ethylene response factor 1), LOX (lipoxygenase), RbohB (respiratory burst oxidase homolog protein B), and two pathogenesis-related (PR) protein genes, PR2b and PR4a, were induced and their expression levels upregulated at 24 or 72 h after transiently expressing ThSCSP_14 (Figure 7A) [30,31,32]. These data showed that ThSCSP_14 is involved in activating immune responses in plants. We also identified the amount of hydrogen peroxide (H_2_O_2_) and callose deposition in *N. benthamiana* leaves after ThSCSP_14 was transiently expressed: at 48 h, H_2_O_2_ had significantly accumulated in those plants undergoing ThSCSP_14 transient expression plants, for which extensive callose deposition was found at 24 h, in comparison with the GFP-only plants (Figure 7B).

## 3. Discussion

Rice kernel smut is a serious disease that poses significant threats to hybrid rice production. However, studies on the virulence functions of effectors in *T. horrida* are few, despite the fact that effectors are a fundamental aspect conferring in phytopathogenic virulence [25,33]. In recent years, based on genome sequencing and structural features, many effectors have been functionally characterized in various plant pathogenic fungi [34,35]. In the *T. horrida* genome, the 575 genes that encode secreted proteins were annotated, of which 103 small cysteine-rich proteins, which are differentially expressed during its infection of hosts, were predicted to be effectors [20]. Through high accuracy screening, Wang et al. reported that the effectors smut_2965 and smut_5844 of *T. horrida* induce cell death in *N. benthamiana* [15].

Tobacco (*N. benthamiana*) is susceptible to a variety of phytopathogens (bacteria, fungi, and so on), making this plant species widely useful in plant–pathogen interaction research, particularly that focused on the activation of innate host immunity and defense signaling [29]. In this study, we used the *N. benthamiana* model system for the systematic screening of *T. horrida* effectors with plant-cell-death-inducing activity, and found that ThSCSP_14 can trigger cell death in *N. benthamiana* from among the selected six candidate effectors. The qRT-PCR results indicated that ThSCSP_14 was upregulated during the early stages of *T. horrida* infection, similar to most other effectors, such as RsIA_NP8 in *Rhizoctonia. solani* AG1-IA, which causes rice sheath blight [36]. Unfortunately, because we were unable to express ThSCSP_14 transiently in rice leaves, we could not test whether ThSCSP_14 could trigger cell death in this host plant species. Furthermore, to clarify the effects of ThSCSP_14 upon its host plants, stable heterologous expression of this gene in rice via genetic transformation methods is needed. In addition, we could treat rice leaves and panicles with proteins coded by ThSCSP_14, and then detect to what extent the defense response is activated [37].

The SP sequence of ThSCSP_14 is necessary to realize its ability of triggered cell death in epidermal cells of *N. benthamiana* leaves. This phenomenon conforms to smut_5844 in *T. horrida*, which also requires its SP sequence to trigger non-host cell death [15]. The same results have been found in two other rice pathogens *U. virens* and *R. solani* AG1-IA [8,36]. Fang et al. reported that eight *U. virens* effectors, UV_44, UV_1338, UV_1423, UV_1533, UV_4753, UV_5517, UV_5851, and UV_6205, which induced cell death in *N. benthamiana* all required their SPs to do so [8]. The effector RsIA_NP8 has been cloned from *R. solani* AG1-IA; its sequence without SP cannot induce the necrosis phenotype in non-host plants [36]. Taken together, this shows that these proteins might function as features secreted to extracellular space [38]. The absence of corresponding SP sequences means that these proteins cannot be secreted out of cells, where these effectors are recognized by extracellular receptors [39]. It might also happen that these effectors are delivered into cells after being secreted, and further recognized by intracellular receptors.

Many effector proteins harbor various conserved domains, such as RxLR and LysM. These are defined as RxLR and LysM effectors, respectively [40,41]. Furthermore, many repeat motifs are also found in effector proteins. Effectors of the rice sheath blight pathogen, *R. solani* AG1 IA, RsIA_NP8, carry RPT motifs [36], and effectors RipG1–RipG7 from *Pseudomonas solanacearum* contain leucine-rich repeats [42]. These conserved motifs play critical roles in the pathogenicity of an effector. For example, the HEAT/armadillo-like repeats of the effector XopN, detected in *Xanthomonas euvesicatoria*, could interact with TFT1, a positive regulator of host immunity [43]. Interestingly, no conserved domains exist in the coding area of ThSCSP_14, so we suspect this protein is a novel effector in plant pathogenic fungi, one that fulfills a special role in the interaction of rice and *T. horrida*. Its action merits further study.

ThSCSP_14 did not trigger cell death in SERK3/Bak1-silenced *N. benthamiana*, showing that it is involved in PTI mediated by cell-surface pattern recognition receptors. Furthermore, SGTI, HSP90, and RAR1 are known to be involved in NLR-mediated ETI responses [27,44], with SGT1 and HSP90 required for plant cell-death triggered by RsIA_NP8 [36]. These three proteins are also related to PTI [45,46]. Here, because cell death was not observed in the SGT1-, HSP90-, and RAR1-silenced *N. benthamiana*, this suggests the ThSCSP_14-induced cell death as related to the defense response is regulated by those three proteins. In synthesizing these results, we speculate that ThSCSP_14 may target a conserved protein or may be directly recognized by an NLR protein in plants. Nevertheless, we cannot rule out the possibility that cell death triggered by ThSCSP_14 is regulated by another, unknown mechanism.

We clarified that the ThSCSP_14 could activate multiple plant defenses, such as the expression of PR genes, the activation of H_2_O_2_, and callose deposition in *N. benthamiana* leaves. ThSCSP_14 induced the expression of LOX and ERF1 at both 24 h and 72 h after inoculation when compared to the control. These two genes contribute to the activity of jasmonic acid and ethylene, and these two hormones-related pathways play key roles in how plants respond to pathogens [31]. The PR genes PR2b and PR4a also underwent upregulated expression in *N. benthamiana* leaves post ThSCSP_14 inoculation, which indicates both two PR genes might be linked to RKS resistance. The burst of H_2_O_2_ and deposition of callose are two typical plant defense responses that happen in the early stages of pathogen infection [47,48]. In our study, ThSCSP_14 triggered the activation of both in *N. benthamiana* leaves, which shows that these two biological processes are important for conferring resistance to *T. horrida*.

## 4. Materials and Methods

### 4.1. Strains, Plant Materials, and Growth Conditions

The *T. horrida* strain JY-521 was saved at the College of Agronomy, Sichuan Agricultural University, and grown on PDA medium (potato 200g, agar 15 g, sucrose 20 g, and distilled water 1000 mL) at 28 °C. *Agrobacterium tumefaciens* GV3101 was cultured on YEP medium (NaCl 5 g, 1% tryptone 10 g, yeast extract 10 g, and distilled water 1000 mL). The yeast strain YTK12 was cultivated on YPDA medium (glucose 20 g, peptone 20 g, yeast extract 10 g, agar 20 g, adenine hemisulfate 0.03 g, and distilled water 1000 mL). Antibiotics and their concentrations were as follows: ampicillin 100 μg/mL^–1^, kanamycin 100 μg/mL^–1^, and rifampin 25 μg/mL^–1^. Rice cultivar 9311A seeds were provided by the College of Agronomy of Sichuan Agricultural University and grown in the field. *Nicotiana benthamiana* plants were grown under a 12-h/12 h night–day photoperiods at 23 °C and 60% relative humidity.

### 4.2. Candidate Effectors

SignalP 4.11 was used to predict the signal peptides of secreted proteins [49], whose transmembrane helices were predicted by the TMHMM Server v 2.02 [50]. We also used the website http://effectorp.csiro.au/ (accessed on 15 May 2022) to predict the effectors; of these, the small encoded proteins (i.e., <500 aa) predicted which contained an N-terminal SP, and lacked a transmembrane domain, were considered candidate effectors [21].

### 4.3. Plasmid Construction of Candidate Effectors

We cloned the full-length or without-signal peptide sequences of candidate effector protein-encoding genes from the *T. horrida* JY-521 strain’s cDNA template using TransStart FastPfu Fly DNA Polymerase (TransGen Biotech, Beijing, China). To construct the transient expression vector, the obtained full length or without-signal peptide of a candidate effector’s coding sequence was linked to the 35S-PMDC32 expression vector, after digestion at BamHI and StuI sites. The restriction enzymes and ClonExpress enzymes (Vazyme Biotech, Nanjing, China) were used according to each product’s description. The primer sequences used in this study are provided in Supplementary Table S1.

### 4.4. Transient Expression Assays

The recombinant 35S-PMDC32 expression vector was introduced into the *A. tumefaciens* strain GV3101. The *A. tumefaciens* strain was cultivated using a fluid lysogeny broth (LB) medium at 28 °C for 48 h with shaking (200 rpm). The strains were collected and then resuspended with MES buffer [15,35]. The suspension with an OD_600_ of 0.6 was incubated for 3 h at room temperature in the dark. The four-leaf stage *N. benthamiana* plants were used for infiltration. The *A. tumefaciens* strain carrying the respective recombinant vector was inoculated into tobacco leaves. The 35S-PMDC32 vector carrying the GFP or Bax gene served as the negative or a positive control, respectively. We infiltrated at least 30 leaves grown on different *N. benthamiana* plants for each treatment, as replicated. The necrosis phenotypes were observed at 4 dpi (days post-inoculation).

### 4.5. Secretory Function Assays of ThSCSP_14′s SP

We investigated the secretory function of ThSCSP_14Δsp using a yeast secretion assay. The sequence of ThSCSP_14Δsp was amplified with designed primers by PCR (Supplementary Table S1) and ligated into the vector pSUC2. The recombinant pSUC2 vector carrying the ThSCSP_14Δsp was transformed into the yeast strain YTK12. Finally, the transformed yeast strain was cultivated on CMD-W, as well as YPRAA plates [15,36].

### 4.6. RNA Extraction and Gene Expression Analysis

Total RNA of the *T. horrida* strain was isolated using Fungal RNA Kit (Omega, Norcross, GA, United States), and that of *N. benthamiana* plants extracted with the Spin Column Plant Total RNA Purification Kit (Sangon Biotech, Shanghai, China). The Transcriptor First Strand cDNA Synthesis Kit (Roche, Shanghai, China) was used to generate the cDNA of *T. horrida* and *N. benthamiana*. The gene expression analysis carried out with the Bio-Rad CFX96 Real-Time PCR System (Bio-Rad, Foster City, CA, USA) according to its instruction manual. The 2^–ΔΔCt^ algorithm method was used to calculate the relative expression levels of the corresponding genes. The relative expression level value of ThSCSP_14 was also calculated by the 2^–ΔΔCt^ method and normalized by the fungal-conserved gene *UBQ* as the reference gene. The relative expression levels of PR genes in *N. benthamiana* were likewise detected using the 2^–ΔΔCt^ method and normalized according to the *Actin* gene (as the reference gene). At least three biological replicates were used. The primer sequences used in this study are provided in Supplementary Table S1.

### 4.7. Western Blot Analysis

The protein extraction and Western blot analysis was implemented as we had described previously [15]. The *N. benthamiana* leaves for protein extraction were collected 72 h after inoculation with *A. tumefaciens*. The extracted proteins were separated via electrophoresis with 12% sodium dodecyl sulfate–polyacrylamide. Proteins were visualized by the eECL Western substrate protocol and observed with X-ray films, and corresponding photographs were taken using an under-the-imaging system.

### 4.8. Trypan Blue Staining, H_2_O_2_ Activity, and Callose Deposition Observation

The necrosis phenotype in epidermal cells of *N. benthamiana* leaves was further verified by trypan blue staining. The *N. benthamiana* leaves at 4 dpi were selected and soaked for 24 h in an aldehyde fixative. Next, the soaked *N. benthamiana* leaves were dyed for 24 h in a boiling trypan blue solution (10% lactic acid, 10% glycerol, 10% ddH_2_O, 10% phenol, and 0.67 g trypan blue), and further decolorized with a chloral hydrate solution (2.5 g/mL) for 3 days [36,51]. The H_2_O_2_ activity in *N. benthamiana* leaves was detected through 3,3′-diaminobenzidine staining, by following a previously reported methodology [52]. Callose deposition in *N. benthamiana* leaves was measured at 1 dpi, as previously described [36]. These experiments were performed at least three times.

### 4.9. Virus-Induced Gene Silencing in N. benthamiana

The *A. tumefaciens* GV3101 strains carrying pTRV2::SOBIR1, pTRV2::RAR1, pTRV2::HSP90, pTRV2::SGT1, pTRV2, and pTRV1 gene were separately grown on YEP plates at 28 °C for 36 h. After the strains were collected, they were resuspended in MES buffer [15,36]. Each pTRV2 and pTRV1 was mixed in a 1:1 ratio with a final OD_600_ of 0.5 for each. The control was pTRV2. The leaves of *N. benthamiana* at the four-leaf stage were infiltrated by the cocultures. The respective silencing efficiency of RAR1, SGT1, HSP90, and SERK3 was analyzed at 21 dpi using qRT-PCR. This test was carried out at least three times. The primer sequences used in this study are listed in Supplementary Table S1.

### 4.10. Subcellular Localization

The sequence of ThSCSP_14 without SP was cloned into the PHB-GFP vector and then transformed into *A. tumefaciens* GV3101. This transformed *A. tumefaciens* GV3101 was allowed to infiltrate *N. benthamiana* leaves according to the methodology described in [52]. Two days after infiltration, subcellular localization was observed using laser confocal fluorescence microscopy.

## 5. Conclusions

We found a secreted protein ThSCSP_14 in the biotrophic fungus *T. horrida*, likely an effector, shown to induce cell death or defense responses in *N. benthamiana* leaves. The predicted SP of this effector is required for its cell-death-inducing ability. The ThSCSP_14 is upregulated by *T. horrida* infection and localizes to the cytoplasm and nucleus of plants. Furthermore, SGT1, RAR1, HSP90, and SERK3 are also required for ThSCSP_14 to trigger cell death in *N. benthamiana*. Our results broaden our understanding of the potential molecular interactive mechanisms of plants and pathogens, while providing valuable timely insight into the pathogenesis mechanism of *T. horrida*. However, the precise molecular mechanisms by which ThSCSP_14 participates in rice–*T. horrida* interactions need to be studied further.

## Data Availability

All datasets generated for this study are included in the article/Supplementary Material.

## Supplementary Materials

The following supporting information can be downloaded at: https://www.mdpi.com/article/10.3390/ijms232315042/s1.
